# Effects of High‐Fructose Corn Syrup Addition to Broiler Diets on Performance, Carcass Yield, Visceral Weights, Gut pH and Some Blood Parameters

**DOI:** 10.1002/vms3.70058

**Published:** 2024-09-26

**Authors:** Gökhan Şen, Mehmet Demirci, Şevket Evci, Ali Şenol, Mehmet Akif Karsli

**Affiliations:** ^1^ Department of Animal Nutrition and Nutritional Diseases, Faculty of Veterinary Medicine Kırıkkale University Kırıkkale Türkiye; ^2^ Department of Plant and Animal Production, Delice VHS Kırıkkale University Kırıkkale Türkiye; ^3^ Department of Biochemistry, Faculty of Veterinary Medicine Kırıkkale University Kırıkkale Türkiye

**Keywords:** antioxidant, biochemical parameters, broiler performance, cytokine, fructose, internal organ

## Abstract

**Background:**

This study hypothesizes that using different amounts of high‐fructose corn syrup (HFCS) in broiler diets may improve performance.

**Objectives:**

This study aimed to determine the effects of HFCS added to broiler diets on performance, cecum pH and some biochemical parameters.

**Methods:**

A total of 120 Ross 308 chicks at the age of 0 day were divided into three main groups with four subgroups each. The groups consisted of a control (CON), low‐HFCS and high‐HFCS groups. The CON group received a diet containing no HFCS, the low‐HFCS diet contained 50 mg/kg HFCS, and the high‐HFCS diet contained 100 mg/kg HFCS. Body weight gain, feed consumption, carcass weight, visceral weight and cecum pH values were examined as performance parameters. Blood samples were taken at the end of the experiment and used to spectrophotometrically determine triglyceride, total cholesterol, high‐density lipoprotein (HDL‐CHO), low‐density lipoprotein (LDL‐CHO), glucose (GLU), creatinine (CRE), uric acid and insulin concentrations, as well as aspartate aminotransferase and alanine aminotransferase activities and oxidative stress markers. Proinflammatory cytokine levels were measured using ELISA test kits.

**Results:**

Feed consumption and body weight gain of the high‐HFCS group decreased (*p* < 0.01). The feed conversion rate was negatively affected in both HFCS groups compared to the CON group (*p* < 0.01). The carcass yields of the groups linearly decreased with the increase of HFCS (*p* < 0.001). Serum LDL cholesterol (*p* < 0.05) and GLU (*p* < 0.01) levels were significantly lower in the HFCS groups than the CON. Serum CRE levels were higher in the low‐HFCS group compared to the other groups (*p* < 0.001). The oxidative stress index (OSI) levels were lower in the low‐HFCS group than the CON group (*p* < 0.05).

**Conclusion:**

The addition of 100 mg/kg HFCS to broiler diets negatively affected performance parameters, but HFCS supplementation positively affected biochemical parameters. In particular, low‐HFCS supplementation decreased the OSI, indicating that it could possibly reduce oxidative stress. Accordingly, HFCS could be added to broiler diets at a level of 50 mg/kg.

## Graphical abstract

The addition of 100 mg/kg high fructose corn syrup (HFCS) to broiler diets negatively affected performance parameters, but HFCS supplementation positively affected biochemical parameters. HFCS could be added to broiler diets at a level of 50 mg/kg.

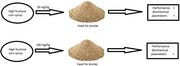

## 1 Introduction

Fructose is found naturally in fruits and vegetables and forms a disaccharide with glucose (GLU) in table sugar. This monosaccharide has been replacing sugar over time and is available as high‐fructose corn syrup (HFCS). Because it is sweeter and cheaper than other sugars commonly used in diets, it is widely used to increase flavour in sugary drinks, baked goods and processed and/or packaged foods (Payant and Chee 2021).

HFCS is obtained as a mixture of GLU and fructose by the enzymatic isomerization of GLU released by the breakdown of corn starch using alpha amylase and glycoamylase enzymes (Khorshidian et al. 2021). After the enzymatic processing, an isomer containing 90% fructose and 10% GLU is obtained and is known as HFCS‐90. HFCS‐90 is mixed with GLU syrup to obtain HFCS‐55 and HFCS‐42. HFCS‐42 is used in foods and canned foods when it is desirable to preserve the natural taste, whereas HFCS‐55 is used in beverages and desserts (Simsek, Topaloğlu, and Dizdar 2021).

The metabolism of fructose in the liver is different from that of GLU. Oral fructose is absorbed from the small intestine and reaches the liver, where it is metabolized through the portal vein. Fructose turns into fructose‐1‐phosphate through the insulin‐independent enzyme fructokinase and undergoes glycolysis, whereas GLU turns into fructose‐1,6‐bisphosphate through the insulin‐dependent enzymes glucokinase and phosphofructokinase, followed by glycolysis (Ouchi et al. 2023).

With increasing HFCS consumption over the years, it was thought that it would eventually replace sucrose over time. However, after increases in the number of obese people, it was determined that there was a positive correlation between HFCS consumption and body weight gain (Sadowska and Rygielska 2019). On the other hand, it has been stated that the intestinal microbiota, which has essential physiological functions in digestion, vitamin synthesis and metabolism, may be associated with obesity because it provides high energy from foods, causes mild inflammation and affects fatty acid tissue composition (Aoun, Darwish, and Hamod 2020). Studies have also shown that fructose‐supplemented diets lead to intestinal microbiome changes and increases in live weight (Shen et al. 2022; Wang, Zhu, et al. 2022).

The health effects of HFCS on rats and mice have been extensively studied (Aslankoç and Özmen 2019; Unsal et al. 2021; Shen et al. 2022; Wang, Zhu, et al. 2022), but studies on its effects in broilers have been very limited (Miles et al. 1987). As HFCS causes weight gain and obesity in mammals, the present study was designed to examine whether it may also be effective in broilers, in which high live weight gain is desired in the shortest time possible. The aim of this study was to investigate the effects of HFCS on broiler performance and health parameters.

## 2 Materials and Methods

### 2.1 Experimental Design and Diets

Experiments were carried out with a total of 120 broiler chicks (Ross 308) for 42 days. The chicks were 0 day old and included both sexes. The chicks were divided into three groups with four subgroups of 10 chicks each. Each subgroup was housed in an area of 1 m^2^. The experimental groups consisted of chicks fed different diets. The control (CON) group was fed a diet no HFCS, whereas the low‐HFCS group was fed a diet containing 50 g/kg HFCS (42% fructose and 2840 kcal/kg energy). The high‐HFCS group was fed a diet containing 100 g/kg HFCS (42% fructose and 2840 kcal/kg energy).

The HFCS diets were supplemented with 42% HFCS, which is commonly used in solid foods (Parker, Salas, and Nwosu 2010). The diets were formulated to be isonitrogenous and isocaloric as starter diets (1–21 days) and grower diets (22–42 days). The ingredients and nutrient compositions of diets are shown in Table 1. The experimental diets were formulated as recommended by the Ross management manual (Anonymous 2014). The diets and water were offered ad libitum. Groups were hosted in an environmentally controlled room that was lit for 24 h with constant led and infrared lighting and heated by electrical heaters. The room temperature was initially set at 35°C and then gradually reduced to 20°C by the sixth week, where it was maintained thereafter.

**TABLE 1:** Ingredients and chemical compositions of rations as wet basis.

Ingredients	Control		Low HFCS		High HFCS	
	Starter (%)	Grower (%)	Starter (%)	Grower (%)	Starter (%)	Grower (%)
Corn	46.25	48.50	47.05	48.85	40.90	42.80
Soybean meal (48% CP)	36.00	32.70	38.30	35.00	39.30	36.30
Wheat bran	9.60	8.70	2.00	1.60	2.00	1.00
HFCS	—	—	5.00	5.00	10.00	10.00
Vegetable oil	3.5	6.00	2.90	5.45	3.05	5.80
Dicalcium phosphate	2.5	2.00	2.50	2.00	2.50	2.00
Limestone	0.90	0.90	1.00	0.90	1.00	0.90
dl‐Methionine	0.40	0.35	0.40	0.35	0.40	0.35
l‐Lysine hydrochloride	0.25	0.25	0.25	0.25	0.25	0.25
l‐Threonine	0.10	0.10	0.10	0.10	0.10	0.10
Salt	0.40	0.40	0.40	0.40	0.40	0.40
Vitamin–mineral premix	0.10	0.10	0.10	0.10	0.10	0.10
Chemical composition						
Dry matter (g/kg)	901.0	900.0	893.0	891.0	884.0	882.0
Crude protein (g/kg)	230.0	210.0	230.0	210.0	230.0	210.0
Crude ash (g/kg)	69.6	64.6	73.7	67.1	106.8	88.2
Crude fibre (g/kg)	54.7	56.7	47.2	49.4	50.9	45.7
ME (kcal/kg)	3027	3160	3002	3132	2976	3117

Abbreviation: CP, Crude protein; HFCS, high‐fructose corn syrup; ME, Metabolizable energy.

### 2.2 Performance

At the end of each week of the experimental period, the chicks and remaining feed were weighed, and the mean body weight gains and feed consumption of every group were determined. Feed conversion rates were calculated weekly as the total feed consumption divided by the body weight gain. At the end of study, 3 chicks from each subgroup were selected (12 chicks from each group), weighed and assessed for carcass yield. After weighing, blood samples were taken, and then the animals were sacrificed.

The head, feet, feathers and internal organs were removed, and the hot carcass weights were immediately recorded. The liver, pancreas, spleen, heart and bursa Fabricius were weighed as well. In addition, after internal organs were removed, samples of cecum content were collected for pH measurement.

### 2.3 Biochemical Analysis

At the end of the 42‐day experiment, blood samples were collected in serum and heparinized tubes from the jugular veins of the chickens. The blood samples were centrifuged at 1600 × *g* for 10 min at 4°C and stored at −80°C. Serum triglyceride (TG), total cholesterol (TCHO), high‐density lipoprotein (HDL‐CHO), low‐density lipoprotein (LDL‐CHO), GLU, creatinine (CRE) and uric acid (UA) concentrations, as well as aspartate aminotransferase (AST) and alanine aminotransferase (ALT) activities, were determined using an AutoAnalyzer (Mindray BS‐400). Serum insulin levels were measured using an ELISA device (Romer, BIO‐TEK ELX800) with commercial ELISA kits (BT‐Lab/EA0012Ch) specific for chickens according to the manufacturer's instructions.

### 2.4 Analysis of Total Oxidant–Antioxidant Status

The total oxidant status (TOS) and total antioxidant status (TAS) were measured in the plasma using commercially available kits (Rel Assay, Turkey) according to Erel (2004, 2005). The TAS results were expressed as micromolar Trolox equivalents per litre, and TOS results were expressed as micromolar hydrogen peroxide equivalents per litre (µmol H_2_O_2_ Eq/L).

### 2.5 Calculation of Oxidative Stress Index (OSI)

The ratio of TOS to TAS was calculated as the OSI. For the calculation, the TAS result was converted to µmol/L, and the OSI value was calculated according to the following equation (Harma, Harma, and Erel 2003; Kösecik et al. 2005; Yumru et al. 2009):

$$\begin{array}{ccc} O\mathrm{SI}\left(a\mathrm{rbit}\mathrm{rary}\;\mathrm{units}\right) & = & T\mathrm{OS}\left(\mu m\mathrm{ol}\;H_{2}O_{2}\;e\mathrm{quiv}\mathrm{alent}/L\right)/\\ & & T\mathrm{AS}\left(\mu m\mathrm{ol}\;T\mathrm{rolox}\;\mathrm{equ}\mathrm{ival}\mathrm{ent}/L\right) \end{array}$$

### 2.6 Cytokine Analysis

Serum levels of tumor necrosis factor‐α (TNF‐α) (BT Lab/EA0010Ch), interleukin‐1β (IL‐1β) (BT Lab/EA0003Ch) and interleukin‐6 (IL‐6)O (BT Lab/EA0004Ch) were measured using an ELISA device (Romer, BIO‐TEK ELX800) and commercial ELISA kits specific for chickens according to the manufacturer's instructions.

### 2.7 Statistical Analysis

All data were analysed using a one‐way analysis of variance (ANOVA) and SPSS 15.0 for Windows. Descriptive statistics were expressed as means and standard errors. Mean values were considered significantly different at *p* < 0.01 and *p* < 0.05. Duncan's multiple range test was performed when the differences among groups were significant.

## 3 Results

The weekly average body weight gains groups are given in Table 2. The average body weight gains were significantly lower in the high‐HFCS group than the CON group (*p* < 0.05) except in the last week. This difference was more obvious in the first 3‐week period (*p* < 0.01). After the third week, the values of the low‐HFCS group started to decrease compared to the CON group. Although this decrease caused a significant weekly difference in the values of the low‐HFCS group compared to the CON group, there was no statistical difference between the groups in the second 3‐week period (*p* > 0.05). The overall average body weight gain of the high‐HFCS group was significantly lower than that of the other groups (*p* < 0.05).

**TABLE 2:** Effects of high‐fructose corn syrup (HFCS) supplementation on average body weight gains of broilers, g/bird (*n*:40).

Weeks	Control	Low HFCS	High HFCS	*p* value
0	43.24 ± 0.10	43.30 ± 0.10	43.60 ± 0.08	0.055
1	138.49 ± 3.68^a^	129.45 ± 1.44^b^	86.03 ± 2.08^c^	<0.001
2	317.61 ± 4.59^a^	320.98 ± 7.43^a^	159.74 ± 8.92^b^	<0.001
3	521.41 ± 5.62^a^	506.28 ± 12.07^a^	260.14 ± 12.99^b^	<0.001
4	552.25 ± 18.54^a^	503.00 ± 15.77^ab^	438.75 ± 32.76^b^	0.024
5	701.50 ± 13.48^a^	631.56 ± 21.92^b^	631.11 ± 18.45^b^	0.036
6	576.25 ± 12.57^a^	520.00 ± 9.87^b^	585.48 ± 6.28^a^	0.009
0–3	977.51 ± 5.05^a^	956.70 ± 15.43^a^	505.90 ± 23.23^b^	<0.001
3–6	1830.00 ± 26.81	1598.50 ± 73.82	1590.36 ± 100.83	0.079
0–6	2807.51 ± 23.39^a^	2555.20 ± 74.48^a^	2096.26 ± 120.89^b^	0.001

*Note*: The difference between values with different letters (a–c) on the same line is important (*p* < 0.05; *p* < 0.01).

The weekly average feed consumption of treatment groups is given in Table 3. There was no difference between the groups in the first and last weeks of the study (*p* > 0.05). However, the average feed consumption of the high‐HFCS group in the other weeks was significantly lower than in the other groups (*p* < 0.05). In the first 3‐week period, the average feed consumption was lowest in the high‐HFCS group and highest in the low‐HFCS group (*p* < 0.01). However, the average feed consumption of the high‐HFCS group was significantly lower overall and in the second 3‐week period than in the other groups (*p* < 0.01).

**TABLE 3:** Effects of high‐fructose corn syrup (HFCS) supplementation on average feed consumptions of experiment broilers, g/bird (*n*:4).

Weeks	Control	Low HFCS	High HFCS	*p* value
1	150.80 ± 8.12	137.53 ± 7.56	130.16 ± 4.06	0.151
2	367.45 ± 4.64^a^	383.76 ± 6.01^a^	248.00 ± 12.06^b^	<0.001
3	463.04 ± 11.51^b^	597.04 ± 15.48^a^	408.88 ± 16.37^c^	<0.001
4	827.60 ± 8.29^a^	809.30 ± 20.87^a^	593.73 ± 36.83^b^	<0.001
5	1021.70 ± 11.73^a^	1011.81 ± 39.28^a^	888.64 ± 40.89^b^	0.036
6	1116.25 ± 13.72	1098.83 ± 39.86	1050.25 ± 29.85	0.360
0–3	981.29 ± 15.61^b^	1118.33 ± 27.67^a^	787.04 ± 27.41^c^	<0.001
3–6	2965.55 ± 20.62^a^	2919.94 ± 94.08^a^	2488.35 ± 120.18^b^	0.008
0–6	3946.84 ± 31.99^a^	4038.26 ± 99.19^a^	3275.38 ± 145.14^b^	0.001

*Note*: The difference between values with different letters (a–c) on the same line is important (*p* < 0.05; *p* < 0.01).

The feed conversion rates of the groups are given in Table 4. The feed conversion rate of the high‐HFCS group was significantly higher in the first weeks (*p* < 0.01) and decreased in the following weeks. The feed conversion rates were similar between the groups in the last week (*p* > 0.05). The feed conversion rate of the high‐HFCS group in the second 3‐week period was similar to that of the CON group and lower than that of the low‐HFCS group (*p* < 0.01). The overall feed conversion rates of the HFCS groups were similar to each other and higher than that of the CON group (*p* < 0.01).

**TABLE 4:** Effects of high‐fructose corn syrup (HFCS) supplementation on feed conversion rates of experiment groups, g/g (*n*:4).

Weeks	Control	Low HFCS	High HFCS	*p* value
1	1.09 ± 0.07^b^	1.06 ± 0.07^b^	1.52 ± 0.07^a^	0.002
2	1.16 ± 0.01^b^	1.20 ± 0.01^b^	1.55 ± 0.02^a^	<0.001
3	0.89 ± 0.01^c^	1.18 ± 0.02^b^	1.58 ± 0.03^a^	<0.001
4	1.50 ± 0.04^a^	1.61 ± 0.01^a^	1.36 ± 0.04^b^	0.002
5	1.46 ± 0.01^b^	1.60 ± 0.03^a^	1.41 ± 0.03^b^	0.001
6	1.94 ± 0.05	2.16 ± 0.05	2.02 ± 0.23	0.621
0–3	1.00 ± 0.02^c^	1.17 ± 0.01^b^	1.56 ± 0.03^a^	<0.001
3–6	1.62 ± 0.02^b^	1.83 ± 0.05^a^	1.57 ± 0.05^b^	0.004
0–6	1.41 ± 0.01^b^	1.58 ± 0.03^a^	1.57 ± 0.04^a^	0.004

*Note*: The difference between values with different letters (a–c) on the same line is important (*p* < 0.05; *p* < 0.01).

Carcass yields, visceral weights and cecum pH values of the experiment groups are shown in Table 5. Both carcass weights and carcass ratios were lowest in high‐HFCS group and the highest in CON group (*p* < 0.01). The liver, spleen, pancreas and bursa Fabricius weights were similar between groups (*p* > 0.05). However, heart weights were higher in the high‐HFCS group and lowest in the low‐HFCS group (*p* < 0.05), but both were similar to the CON group. There was no significant difference between the cecum pH values of the groups (*p* > 0.05).

**TABLE 5:** Effects of high‐fructose corn syrup (HFCS) supplementation on carcass yields, visceral weights and cecum pH values of broilers (*n*:12).

	Control	Low HFCS	High HFCS	*p* value
Carcass weight, g	2255.25 ± 37.50^a^	1996.42 ± 24.52^b^	1676.17 ± 44.67^c^	<0.001
Carcass rate, %	77.83 ± 0.36^a^	75.00 ± 0.52^b^	73.16 ± 0.43^c^	<0.001
Liver, g	48.27 ± 2.10	44.21 ± 1.57	46.30 ± 1.83	0.310
Heart, g	10.78 ± 0.50^ab^	10.19 ± 0.39^b^	12.24 ± 0.65^a^	0.027
Pancreas, g	4.29 ± 0.14	3.91 ± 0.21	4.02 ± 0.12	0.255
Bursa Fabricius, g	5.24 ± 0.68	3.78 ± 0.39	4.34 ± 0.36	0.125
Spleen, g	2.09 ± 0.22	2.48 ± 0.21	1.96 ± 0.23	0.224
Cecum pH	7.74 ± 0.10	7.63 ± 0.10	7.49 ± 0.07	0.187

*Note*: The difference between values with different letters (a–c) on the same line is important (*p* < 0.05; *p* < 0.01).

The serum biochemistry results are shown in Table 6. TG, TCHO, HDL‐CHO, LDL‐CHO, GLU and insulin levels were lower in the HFCS groups than the CON group. Although LDL‐CHO (*p* < 0.01) and GLU (*p* < 0.01) levels of the HFCS groups were similar, they were significantly lower than that of the CON group (*p* < 0.01). The serum CRE level of the high‐HFCS group was similar to that of the CON, whereas that of the low‐HFCS group’ was higher than that of the CON group (*p* < 0.01). AST and ALT activities and CRE and UA levels were higher in the HFCS groups than the CON group.

**TABLE 6:** Effects of high‐fructose corn syrup (HFCS) supplementation on some blood parameter values of broilers (*n*:12).

	Control	Low HFCS	High HFCS	*p* value
AST (U/L)	269.85 ± 40.15	310.06 ± 24.32	337.26 ± 17.41	0.256
GLU (mg/dL)	202.30 ± 3.32^a^	174.00 ± 5.66^b^	179.58 ± 5.31^b^	0.001
TCHO (mg/dL)	150.56 ± 7.34	131.50 ± 5.56	147.25 ± 7.31	0.117
HDL‐CHO (mg/dL)	101.75 ± 4.28	93.73 ± 3.81	96.69 ± 3.56	0.353
LDL‐CHO (mg/dL)	37.50 ± 2.65^a^	28.67 ± 1.61^b^	28.73 ± 1.93^b^	0.007
TG (mg/dL)	23.00 ± 1.95	20.17 ± 1.64	24.36 ± 2.39	0.322
ALT (U/L)	40.39 ± 5.81	44.42 ± 4.92	43.08 ± 4.34	0.853
UA (mg/dL)	2.82 ± 0.40	3.97 ± 0.47	3.70 ± 0.37	0.154
Insulin (µIU/mL)	4.21 ± 0.15	3.86 ± 0.10	3.91 ± 0.12	0.127
CRE (mg/dL)	0.32 ± 0.01^b^	0.41 ± 0.01^a^	0.34 ± 0.02^b^	<0.001

*Note*: The difference between values with different letters (a–b) on the same line is important (*p* < 0.05; *p* < 0.01).

Abbreviations: ALT, alanine aminotransferase; AST, aspartate aminotransferase; CRE, creatinine; GLU, glucose; HDL‐CHO, high‐density lipoprotein cholesterol; LDL‐CHO, low‐density lipoprotein cholesterol; TCHO, total cholesterol; TG, triglyceride; UA, uric acid.

The results of the oxidative stress parameters from the plasma are given in Table 7. In the HFCS groups, an increase in plasma TAS levels and decrease in plasma TOS levels were observed compared to the CON group, but the differences were not statistically significant (*p* > 0.05). The OSI value was lower in the low‐HFCS group than in the CON group (*p* < 0.05) and similar to that of the high‐HFCS group. The results of the serum cytokine levels are given in Table 8. The pro‐inflammatory cytokine levels showed an increase in the HFCS groups compared to the CON group, but the difference was not statistically significant (*p* > 0.05).

**TABLE 7:** Effects of high‐fructose corn syrup (HFCS) supplementation on oxidative stress parameters values of broilers (*n*:12).

	Control	Low HFCS	High HFCS	*p* value
TAS (mmol/L)	1.89 ± 0.10	1.94 ± 0.07	2.00 ± 0.07	0.598
TOS (µmol/L)	6.57 ± 0.44	5.12 ± 0.68	5.79 ± 0.52	0.190
OSI (arbitrary unit)	0.36 ± 0.02^a^	0.25 ± 0.03^b^	0.32 ± 0.03^ab^	0.047

*Note*: The difference between values with different letters (a–b) on the same line is important (*p* < 0.05).

Abbreviations: OSI, oxidative stress index; TAS, total antioxidant status; TOS, total oxidant status.

**TABLE 8:** Effects of high‐fructose corn syrup (HFCS) supplementation on cytokine levels of broilers (*n*:12).

	Control	Low HFCS	High HFCS	*p* value
IL‐6 (ng/L)	109.24 ± 22.27	139.80 ± 17.15	108.96 ± 17.05	0.415
TNF‐α (ng/L)	37.43 ± 4.65	41.03 ± 4.11	50.05 ± 3.86	0.116
IL‐1β (ng/L)	47.17 ± 5.09	62.19 ± 4.69	56.59 ± 4.28	0.089

## 4 Discussion

The goal in broiler feeding is to obtain the highest carcass yield with the least feed. Even though weekly body weight gain varied with the addition of HFCS, in the high‐HFCS group, body weight gain significantly decreased both in the first 3‐week period and overall. In a study conducted by Hussein et al. (2016), the body weight gain results were similar to those of the present study. The body weight gain of the group consuming feed containing 5% sugar syrup in the overall period was similar to that of the CON, but it decreased in the group consuming feed containing 10% sugar syrup. On the other hand, fructose metabolism has lipogenic properties, it takes place in the liver, and it affects the production of components necessary for TG synthesis. It is not affected by phosphofructokinase, which means that TG production increases rapidly with high levels of fructose consumption (Angelopoulos et al. 2009).

According to Bocarsly et al. (2010), among rats that had access to HFCS and sucrose, those that had access to HFCS had higher weight gain than those with access to sucrose even though the HFCS group was ingesting fewer calories. In addition, the fat pads (an obesity indicator) and serum TG levels were higher in the HFCS groups of male rats over a 6‐month period. But TG values in the present study were no different between groups. This result may be due to the current study period being limited to 6 weeks. Therefore, the body weight gain that we expected for broilers with HFCS did not occur with short‐term exposure.

Body weight gain is expected not only in obesity‐related adiposity, but also in muscle and other tissues. But in the present study, the carcass weight and carcass ratio also showed a linear change with HFCS, and visceral weights were similar to that of the CON group. Similar to body weight gain, the feed consumption was low in the overall period. It has been argued that unlike GLU, fructose does not stimulate the release of insulin and leptin (Sadowska and Rygielska 2019). Although HFCS was expected to stimulate hunger due to its low leptin‐release effect in the present study, the decrease in feed consumption of the HFCS groups may be due to the fact that the HFCS used contains 42% fructose and higher GLU levels.

Sadowska and Rygielska (2019) conducted a study with rats fed feeds containing sugar or HFCS at 10%. They stated that the lower feed consumption of the group that consumed the sugar‐containing feed was due to the increase in leptin, which stimulates the satiety centre. In the same study, the insulin level was similar between the groups, as in the present study. M'Sadeq et al. (2015) reported an increase in feed consumption of broilers fed diets containing resistant starch, which may have been due to less energy intake resulting from poor digestion and compensation for this energy deficiency. Lotfi et al. (2019) stated that the feed consumption of a group consuming feed containing 4% resistant starch was higher than that of a group consuming feed containing fructooligosaccharide, whereas the body weights of the groups were similar in the overall period.

After the separation of fructans into their monomers by bacterial oxidases, fermentation into short‐chain fatty acids occurs. Acetic acid provides energy to peripheral tissues, and butyric acid is used as an energy source by colonocytes. Propionic acid is used for gluconeogenesis in the liver (Bogusławska‐Tryk, Piotrowska, and Burlikowska 2012). It can be assumed that feed consumption decreased as a result of providing the required energy in lower amounts in the HFCS groups. However, the similar body weight‐gain reductions and organ and tissue weight gains to the CON indicate that the conversion of feed in the HFCS groups was low. The feed conversion rates of the HFCS groups also showed this. The reason why the high‐HFCS group consumed less than the low‐HFCS group may be that the pellets could not be broken apart and consumed due to the higher binding effect of the HFCS, and the high‐HFCS feed had twice as much HFCS.

Intestinal health is an important factor that affects both the health and growth performance of monogastric animals. The type, density and source of carbohydrates in the diet affect the intestinal microbiota. Short‐chain fatty acids are released as a result of fermentation of carbohydrate fractions by anaerobic bacteria (Adebowale et al. 2019). Ndelekwute, Unah, and Udoh (2019) determined that different organic acids have a positive effect on acidification of intestine. However, in the present study, the difference between the pH values of the groups was not significant.

Many studies have shown that high‐fructose diets increase TG concentrations in animals and humans and that simple sugars cause fasting hypertriglyceridemia by stimulating hepatic de novo lipogenesis (Le and Tappy 2006; Havel 2005; Bizeau and Pagliassotti 2005). Lowndes et al. (2014) stated that increased carbohydrate intake promotes the formation of nascent very low‐density lipoprotein (VLDL) particles by combining glycerol, free fatty acids and Apo B, thus increasing TG levels. Wang et al. (2014) reported a systematic meta‐analysis on the basis of various studies investigating the chronic effect of replacing isocaloric or hypercaloric oral fructose with a reference carbohydrate on postprandial TGs in humans. Fructose had a significant postprandial TG‐raising effect under hypercaloric feeding conditions supplemented with high doses of fructose (∼+175 g/day) and excess energy (+25% energy), and the increase in TG levels was approximately 0.81 mmol/L.

In a study conducted on rats, Gun et al. (2016) reported that when HFCS was mixed with drinking water at a concentration of 30% for 6 weeks, there was an increase in TG, TCHO, HDL‐CHO, LDL‐CHO, GLU and UA levels compared to the CON group. Chong, Fielding, and Frayn (2007) showed that eating a meal with fructose, which is abundant in the diet of HFCS‐fed animals, reduces the activity of lipoprotein lipase, and impaired catabolism of TG (present in VLDL) hinders fat‐tissue synthesis. A number of studies have shown that increased fructose consumption significantly increases the rate of lipogenesis, accelerates the esterification of fatty acids and promotes VLDL secretion (Havel 2005; Bizeau and Pagliassotti 2005; Lowndes et al. 2014). The effect increases with increasing dietary fructose.

In the present study, there were decreases in lipid parameters in the HFCS groups compared to the CON group, but only the decrease in LDL‐CHO levels was significant. This is contrary to the results reported by Lowndes et al. (2014), Wang et al. (2014) and Gun et al. (2016). The lack of hyperlipidaemia observed in HFCS‐supplemented groups may be due to the use of broiler chickens, the net energy capacity of the ration and the isocaloric adjustment of the diets given to the groups.

Fructose is absorbed in the small intestine after consumption and transported through the portal vein to the liver, where it is metabolized. Fructose's metabolism stages in the liver are different from those of GLU. It is converted into fructose‐1‐phosphate by the enzyme fructokinase, independently of insulin and acutely increases glucokinase activity (Le and Tappy 2006; Vila et al. 2011; Quchi et al. 2023). Fructose consumption can be expected to reduce glycemia in hyperglycaemic patients as it increases glucokinase activity, which plays an important role in the CON of GLU homeostasis and phosphorylation of glucose to glucose‐6‐phosphate, facilitating its storage as glycogen and excretion through glycolysis (Le and Tappy 2006).

In the present study, the decrease in GLU levels despite 6 weeks of exposure to HFCS is consistent with the findings of Gaby (2005) and Collino et al. (2010). They stated that short‐term and/or low consumption of fructose with increased glucokinase activity may have a regulating effect on blood GLU levels, whereas long‐term exposure may increase the levels. During the metabolism of fructose in the liver, the amount of intracellular adenosine triphosphate (ATP) decreases, and adenosine monophosphate (AMP) is eventually converted to UA (Madero et al. 2011; Sánchez‐Lozada et al. 2008). After intake of fructose, which is the only sugar that increases UA levels, UA concentration increases in both the cells and in the blood within approximately 30–60 min (Madero et al. 2011; Kaplan, Bulut, and Mir 2011). In this study, the increase in UA levels in the HFCS groups compared to the CON group can probably be explained by the fructose competing with UA during renal excretion and reducing UA excretion (Johnson et al. 2007; Kaplan, Bulut, and Mir 2011).

In addition, it is known that UA contributes to the body's defence systems and may have an effect on various factors, such as decrease in glomerular filtration, renal hypoperfusion, increased tubular reabsorption or decreased excretion (Johnson et al. 2005; Madero et al. 2009; Yılmaz 2021). Long‐term increases in the amount of UA, which normally occurs as the end product of purine metabolism, may be associated with tissue damage in the urinary system and the progression of chronic kidney disease (Edwards 2008; Lapsia et al. 2012; Hahn et al. 2017).

The CRE test is the preferred test for diagnosing kidney disease (Mert and Mert 2023). Andres‐Hernando et al. (2023) stated that HFCS (55%) added to the drinking water of normal and obese mice significantly increased blood urea nitrogen (BUN) and plasma CRE levels, caused chronic kidney damage and increased mortality. In obese mice, the survival rate decreased, and kidney damage increased. In the present study, serum CRE (*p* < 0.001) and UA (*p* > 0.05) levels increased in the HFCS groups compared to the CON group, which may be due to a pronounced sensitivity to fructose. This was indicated by higher expression of the fructose transporter (Glut5) due to kidney damage and higher ketohexokinase (KHK) activity associated with higher fructose absorption (Andres‐Hernando et al. 2023).

Transaminase enzymes (ALT and AST) play an important role in the diagnosis of liver disorders resulting from various toxicities and infections and are used as markers of cell damage (Kew 2000; Nelson and Cox 2005). In a study conducted in male rats, an increase in ALT activities was reported when 15% HFCS solution was given 3 days a week (Figlewicz et al. 2009). In a study in which a high‐fat/high‐fructose diet was applied, hepatic steatosis and an increase in ALT and AST activities were observed (Collino et al. 2010).

According to the literature, it has been determined that HFCS exposure at various rates adversely affects the liver tissue in which it is metabolized, as well as sinusoidal degeneration, oedema and mononuclear cell infiltration according to histopathological examinations. Increases in AST and ALT enzymatic activities were also observed (Collino et al. 2010; Turasan 2014). It is thought that the increase observed in serum AST and ALT activities in the HFCS group may have been due to the possible harmful effects of HFCS on liver tissue.

The liver is located in the metabolic centre of the body and is a very important organ for neutralizing toxic substances and removing them from the body. Various studies have reported that oxidative damage plays an important role in liver disorders (Alkadi 2018; Cao et al. 2022). Unsal et al. (2021) stated that 15% HFCS given orally to rats for 12 weeks decreased the antioxidant parameters superoxide dismutase (SOD), glutathione (GSH) and glutathione peroxidase (GSH‐Px) activities in liver tissue homogenates and serum samples, whereas it increased the oxidant parameters malondialdehyde (MDA) and nitric oxide (NO). In this study, it was surprising that TAS levels increased and TOS levels decreased in the HFCS groups compared to the CON group, which is contrary to the findings of Unsal et al. (2021).

In studies conducted to evaluate the oxidant–antioxidant profile, the OSI was used in addition to TAS and TOS levels to monitor changes as a result of oxidative stress. The OSI is used as a two‐way indicator of oxidant–antioxidant balance in oxidative stress (Savaş 2019). In the present study, the calculated OSI values were similar in HFCS groups but decreased compared to the CON group. The HFCS caused an increase in TAS levels (*p* > 0.05) and a decrease in TOS (*p* > 0.05) and OSI (*p* < 0.05) levels, which suggests that HFCS may have a reducing effect on oxidative damage in the short term and at the given doses (50 and 100 g/kg).

Experimental studies reported that fructose exposure activates cellular stress responses in various tissues and causes inflammation and lipid accumulation (Le and Tappy 2006; Ma et al. 2015). TNF‐α, IL‐1 and IL‐6 are important basic proinflammatory cytokines used to evaluate the inflammatory response and are induced by high TG concentrations associated with insulin resistance (Le and Tappy 2006; Bidwell 2017). Wang, Ji, et al. (2022) stated that 25% HFCS added to the drinking water of mice promoted proinflammatory macrophage activation via reactive oxygen species (ROS)‐mediated NF‐kB signalling. Furthermore, serum TNF‐α, IL‐1 and IL‐6 levels increased in HFCS‐receiving groups compared to the CON group. They examined an experimental dextran sulphate sodium (DDS)‐induced mouse colitis model (Wang, Ji, et al. 2022). They stated that increases in proinflammatory cytokine levels may support the immune response and that targeting other proinflammatory cytokines may be an effective way to prevent or CON HFCS‐exacerbated colitis.

In the present study, an increase in proinflammatory cytokines was observed in the HFCS groups compared to the CON group, similar to Wang, Ji, et al. (2022), although the result was not statistically significant (*p* > 0.05). It has been stated that increases in proinflammatory cytokine levels increase neutrophil accumulation and inflammatory responses (Zhang et al. 2018; Ayaz 2018). Nevertheless, more studies are needed to determine the possible effects of HFCS exposure at different doses and durations on this mechanism.

## 5 Conclusion

The data showed that the addition of 100 mg/kg HFCS to broiler diets negatively affected performance parameters. HFCS supplementation positively affected biochemical parameters. In particular, low‐HFCS supplementation decreased GLU and LDL cholesterol levels. In addition, low‐HFCS supplementation decreased the OSI, indicating that it could reduce possible oxidative stress.

Short‐term fructose use may play a regulatory role in blood GLU levels and may have radical scavenging and lipid peroxidation inhibitory roles with the effect of increased UA levels due to exposure. However, oxidative stress, inflammation and tissue damage may also be observed in proportion to the increased amount and duration of exposure. Accordingly, HFCS can be added to broiler diets at a level of 50 mg/kg. This evaluation should be supported by new studies that are planned from a broader perspective on broiler. Additionally, a long‐term feeding trial could be useful to test the use of HFCS when preparing diets for chickens.

## Author Contributions

**Gökhan Şen**, **Ali Şenol** and **Mehmet Akif Karsli**: conceptualization and methodology. **Gökhan Şen**, **Mehmet Demirci**, **Şevket Evci** and **Mehmet Akif Karsli**: software and data curation. **Gökhan Şen**, **Mehmet Demirci**, **Şevket Evci** and **Ali Şenol**: investigation. **Mehmet Akif Karsli**: validation. **Gökhan Şen** and **Ali Şenol**: formal analysis. **Gökhan Şen** and **Mehmet Akif Karsli**: supervision. **Gökhan Şen**: funding acquisition. **Gökhan Şen**: visualization and project administration. **Gökhan Şen** and **Ali Şenol**: resources and writing – original draft. **Gökhan Şen**, **Ali Şenol** and **Mehmet Akif Karsli**: writing – review and editing.

## Ethics Statement

The experimental procedures were approved with the 2020/06 meeting and decision number 36 by the Kırıkkale University Animal Experiments Local Ethics Committee.

## Conflicts of Interest

The authors declare no conflicts of interest.

### Peer Review

The peer review history for this article is available at https://publons.com/publon/10.1002/vms3.70058.

## Data Availability

The data that support the findings of this study are available from the corresponding author upon reasonable request.
